# In Vitro Drug Screening Using iPSC-Derived Cardiomyocytes of a Long QT-Syndrome Patient Carrying KCNQ1 & TRPM4 Dual Mutation: An Experimental Personalized Treatment

**DOI:** 10.3390/cells11162495

**Published:** 2022-08-11

**Authors:** Feifei Wang, Yafan Han, Wanyue Sang, Lu Wang, Xiaoyan Liang, Liang Wang, Qiang Xing, Yankai Guo, Jianghua Zhang, Ling Zhang, Tuerhong Zukela, Jiasuoer Xiaokereti, Yanmei Lu, Xianhui Zhou, Baopeng Tang, Yaodong Li

**Affiliations:** 1 Cardiac Pacing and Electrophysiology Department, The First Affiliated Hospital of Xinjiang Medical University, Urumqi 830000, China; 2 Xinjiang Key Laboratory of Cardiac Electrophysiology and Cardiac Remodeling, The First Affiliated Hospital of Xinjiang Medical University, Urumqi 830000, China; 3 Xinjiang First Aid Center, People’s Hospital of Xinjiang Uygur Autonomous Region, Urumqi 830000, China

**Keywords:** long QT syndrome, arrhythmia, induced pluripotent stem cell, cardiomyocytes, directed differentiation, disease model, drug screening

## Abstract

Congenital long QT syndrome is a type of inherited cardiovascular disorder characterized by prolonged QT interval. Patient often suffer from syncopal episodes, electrocardiographic abnormalities and life-threatening arrhythmia. Given the complexity of the root cause of the disease, a combination of clinical diagnosis and drug screening using patient-derived cardiomyocytes represents a more effective way to identify potential cures. We identified a long QT syndrome patient carrying a heterozygous KCNQ1 c.656G>A mutation and a heterozygous TRPM4 c.479C>T mutation. Implantation of implantable cardioverter defibrillator in combination with conventional medication demonstrated limited success in ameliorating long-QT-syndrome-related symptoms. Frequent defibrillator discharge also caused deterioration of patient quality of life. Aiming to identify better therapeutic agents and treatment strategy, we established a patient-specific iPSC line carrying the dual mutations and differentiated these patient-specific iPSCs into cardiomyocytes. We discovered that both verapamil and lidocaine substantially shortened the QT interval of the long QT syndrome patient-specific cardiomyocytes. Verapamil treatment was successful in reducing defibrillator discharge frequency of the KCNQ1/TRPM4 dual mutation patient. These results suggested that verapamil and lidocaine could be alternative therapeutic agents for long QT syndrome patients that do not respond well to conventional treatments. In conclusion, our approach indicated the usefulness of the in vitro disease model based on patient-specific iPSCs in identifying pharmacological mechanisms and drug screening. The long QT patient-specific iPSC line carrying KCNQ1/TRPM4 dual mutations also represents a tool for further understanding long QT syndrome pathogenesis.

## 1. Introduction

Long QT syndrome (LQTS) is a type of cardiovascular disease characterized by prolonged QT interval and malignant arrhythmia [1,2,3]. Patients diagnosed with long QT syndrome suffer from symptoms including syncopal episodes, palpitations and electrocardiographic abnormalities and are at risk of sudden death [1,2,3]. The prevalence of congenital long QT syndrome is estimated to be close to 1 in 2000 [4], which accounts for approximately 75% of all LQTS cases [3]. Beta-blocker therapy is widely used in the management of LQTS [5]. However, the efficacy of beta-blocker varies among patients with different types of LQTS and events such as syncope, aborted cardiac arrest and LQTS-related deaths may continue to occur [6,7,8]. Implantable cardioverter-defibrillator (ICD) may prevent sudden cardiac deaths, but at the same time it introduces serious complications and has negative impacts on patient’s overall quality of life [9,10,11]. Therefore, LQTS remains an unmet medical need awaiting more effective treatment.

Recent advancement in identifying the genes and corresponding mutations related to congenital LQTS improved our understanding of the disease significantly. Differences in gene mutations might result in variations in pathogenesis and therefore clinical heterogeneity in LQTS patients, causing complications and reduced effectiveness in treatments [7,12,13,14]. Such a situation highlights the necessity of understanding the mechanisms underlining LQTS manifestation of each known correlated gene mutation, which could facilitate the development of corresponding treatment strategies.

Most LQTS cases could be categorized into three subgroups, namely LQT1, LQT2 and LQT3 [13,14]. LQT1 is predominately correlated to mutations of the KCNQ1 gene, while LQT2 and LQT3 are correlated to mutations of the KCNH2 gene and SCN5A gene, respectively [15,16]. Existing in vitro and animal models of LQTS are unable to mimic the unique electrophysiological characteristics of human cardiomyocytes [17,18]. We therefore switched to establishing a human-cardiomyocyte-based disease model for the purpose of more efficient drug screening.

The current study was based on an LQTS patient who received conventional diagnosis and treatments for over 5 years with limited benefits. The patient suffered not only from typical LQTS symptoms including palpitations, seizures and chest tightness, but also more dangerous ones including potentially life-threatening ventricular fibrillation. Treatment with the use of beta-blockers was found not to be effective, while ICD implantation in combination with anti-arrhythmia medications barely managed the symptoms. However, ICD discharge happened frequently, which caused deterioration of patient quality of life. Given the critical condition of the patient, identification of better alternative therapeutic agents is the key towards a better clinical outcome. Considering the potentially complicated causality underlining the LQTS symptoms of the patient, we decided to employ induced pluripotent stem cell technology to reprogram somatic cells of the LQTS patient into iPSCs to act as a personalized disease modelling platform. Sequencing data suggested that the patient is carrying not only heterozygous KCNQ1 c.656G>A mutation but also a heterozygous TRPM4 c.479C>T mutation. Mutations in the TRPM4 gene have been found to be related to various cardiovascular conditions including complete heart block, ventricular tachycardia and Brugada syndrome [19,20,21,22]. Aiming to identify better therapeutic agents for the patient as soon as possible, we differentiated LQTS patient-specific iPSCs into cardiomyocytes for drug screening. Electrophysiological analysis indicated that cardiomyocytes derived from LQTS patient-specific iPSCs demonstrated QT-intervals significantly longer than cardiomyocytes derived from health donor iPSCs. Based on literature, we selected verapamil and lidocaine as alternative therapeutic agents. Verapamil is a calcium channel blocker primarily used to treat high blood pressure, chest pain caused by angina and arrhythmia [23]. Lidocaine is a sodium channel blocker used as an arrhythmia drug, as well as an anesthetic for local or intravenous administration [24,25]. Both drugs are known to shorten QT interval in individuals who do not have LQTS and are not considered mainstream medication for treating LQTS [26,27]. Treatment of 1 μM verapamil or 30 μM lidocaine significantly reduced QT-intervals of patient-specific cardiomyocytes to a level comparable to that of healthy donor cardiomyocytes. Addition of verapamil to the treatment regime successfully reduced ICD discharge frequency.

In comparison to the 5-year-long conventional diagnosis and treatment with limited success, the current study derived LQTS patient-specific, KCNQ1 & TRPM4 dual mutation cardiomyocytes to act as a disease model for drug screening. Two potential alternative therapeutic agents were identified in less than 1.5 years since iPSC reprogramming started. By adding verapamil to the treatment regime, ICD discharging frequency was reduced. ECG also demonstrated reduction of QT interval, indicating that the in vitro effect of verapamil was reflected in vivo. Our results indicated that patient-specific iPSCs and cardiomyocytes represent efficient disease models for screening alternative therapeutic agents for congenital LQTS even for patients with a complicated genetic background.

## 2. Materials and Methods

### 2.1. Patient Characteristics

The patient demonstrated not only typical characteristics of long QT syndrome including fainting, seizures, shortness of breath and palpitations, but also electrical storms and cardiac arrest. Patient symptoms, diagnosis and treatment history are listed in Table S1. Peripheral blood of the patient was collected for PBMC isolation and subsequent reprogramming into LQTS patient-specific iPSCs (LQTS-iPSCs). Peripheral blood of a healthy donor was used to generate control iPSCs (Ctrl-iPSCs). The study was performed in accordance with the declaration of Helsinki, and all protocols were approved by the host institutional review boards.

### 2.2. PBMC Isolation and Reprogramming into iPSCs

PBMCs were isolated from (i) LQTS patient and (ii) healthy donors by centrifugation using Vacutainer CPT (BD Biosciences, Franklin Lakes, NJ, USA), cultured for 3~5 days and subsequently transduced with CytoTune-iPS 2.0 Sendai Reprogramming Kit (ThermoFisher Scientific, Waltham, MA, USA) following manufacturer’s instructions. At 24~48 h after transduction, cells were replated onto 10 cm culture dishes coated with Matrigel (Corning, Corning, NY, USA) and maintained in mTeSR1 medium (STEMCELL Technologies, Vancouver, BC, Canada). iPSC colonies were picked at day 20~30 after transduction and expanded in mTeSR1 medium. Cells were passaged upon reaching 50~70% confluence at a ratio of 1:4~1:8 by using ACCUTASE (ThermoFisher Scientific, Waltham, MA, USA). Cells were maintained at 37 °C and 5% CO_2_ in a humidified incubator. Clearance of reprogramming vector was confirmed with PCR. Throughout the study, iPSCs were cultured in feeder-free conditions. Established iPSC cultures were analyzed under light microscope (Olympus, Shinjuku, Tokyo, Japan) to confirm their morphology. iPSC cultures were further analyzed for expressions of pluripotent markers to confirm their identity and purity. iPSC cultures were also subjected to trilineage differentiation with the use of STEMdiff™ Trilineage Differentiation Kit (STEMCELL Technologies, Vancouver, BC, Canada) following manufacturer’s instructions to confirm their differentiation potential. Finally, LQTS-iPSCs were further analyzed using whole exome sequencing to confirm the existence of KCNQ1 and TRPM4 mutations. Please refer to subsequent sections for details.

### 2.3. Cardiomyocyte Differentiation

Ctrl-iPSCs and LQTS-iPSCs were cultured in 6-well plates coated with Matrigel (Corning, Corning, NY, USA) using mTeSR1 medium. Upon reaching 90% confluency, Ctrl-iPSCs and LQTS-iPSCs cultures were subjected to cardiomyocytes differentiation. In brief, on day 0 and day 1, mTeSR1 medium was removed and iPSC cultures were given #1 differentiation medium (RPMI 1640 with 2% B27 supplement minus insulin, both from ThermoFisher Scientific, Waltham, MA, USA) supplemented with 6 μM CHIR-9902 (Sigma-Aldrich, Burlington, MA, USA). Medium change was performed every 24 h. CHIR-99021 is a selective inhibitor of glycogen synthase kinase 3-β that activates the canonical Wnt signaling pathway. On day 2, culture medium was changed to #1 medium without CHIR99021. On day 3 and day 4, culture medium was changed to #1 medium with 5 μM of IWR-1 (Sigma-Aldrich, Burlington, MA, USA), which is a Wnt antagonist. Medium change was performed every 24 h. On day 5 to 8, the medium was replaced with #1 medium without CHIR-99021 or IWR-1. Medium change was performed every 24 h. On day 9, the medium was replaced with maintenance medium (RPMI 1640, 2% B27 supplement and 2% FBS, all from ThermoFisher Scientific, Waltham, MA, USA) and medium change was performed every other day. Usually, the cells began to demonstrate spontaneous contractions on day 8 to 10 of differentiation. At 3 to 4 days after spontaneous contraction was observed, cardiomyocyte cultures were subjected to purification as described by Tohyama et al. with the use of glucose-free culture medium [28]. After 4 days of purification, culture medium was switched back to maintenance medium and maintained for another 1 to 2 days. At day 16–20 since differentiation started, purified cardiomyocytes were dissociated by incubation at 37 °C in 0.25% trypsin EDTA (ThermoFisher Scientific, Waltham, MA, USA) followed by centrifugation at 300 g for 5 min. Cardiomyocytes were resuspended at 1 × 10^7^ cells per mL in Cryostor CS10 (BioLife Solutions, Bothell, WA, USA) in cryogenic vials (Corning, Corning, NY, USA). Cardiomyocytes derived from Ctrl-iPSCs were regarded as control cardiomyocytes (Ctrl-CMs), while cardiomyocytes derived from LQTS-iPSCs were regarded as LQTS cardiomyocytes (LQTS-CMs). Cryopreserved cardiomyocytes were thawed in a 37 °C water bath and cultured in maintenance medium before being subjected to characterization and other experiments. As for cardiomyocyte characterization, thawed cardiomyocytes were subjected to flow cytometry analysis to ensure purity as determined by proportion (in %) of cells positive for cardiomyocyte marker cardiac troponin T (cTnT). Thawed cardiomyocytes were also cultured on glass cover slips coated with Matrigel (Corning, Corning, NY, USA) in maintenance medium and subsequently subjected to immunofluorescence analysis to evaluate the expression of cardiomyocyte markers cTnT and α-actinin. Please refer to subsequent Section 2.5, Immunofluorescence and Section 2.6, Flow Cytometry for details.

### 2.4. Drug Treatment and Electrophysiological Examination

Thawed Ctrl-CMs and LQTS-CMs were cultured on glass cover slips coated with Matrigel (Corning, Corning, NY, USA) in maintenance medium (RPMI 1640 with 2% B27 supplement and 2% FBS). When cardiomyocytes were being subjected to drug treatment and manual patch clamp recording, maintenance medium was replaced by patch clamp external solution. Given that cardiomyocyte culture derived from iPSC might consist of ventricular, atrial and nodal cells, only cells that demonstrated the typical ventricular action potential pattern were used for data collection during patch clamp recording. Verapamil (1 mM) and lidocaine (30 mM) stock solutions were prepared by first dissolving the chemicals (Tocris Bioscience, Bristol, UK) in DMSO (Sigma-Aldrich, Burlington, MA, USA). Stock solutions were added to patch clamp external solution during drug treatment to reach working concentrations of 1 μM (verapamil) and 30 μM (lidocaine), respectively. Concentrations of verapamil and lidocaine were determined based on literature [29,30]. Manual whole cell patch clamp (current clamp) was performed with the use of EPC-10 patch clamp amplifier (HEKA Electronics, Lambrecht (Pfalz), Germany). Glass pipettes used for patch clamp recording were prepared by P-97 Micropipette Puller (Sutter Instrument, Novato, CA, USA). Data acquired were analyzed with the use of HEKA Patchmaster (V2x73.2) and Clamfit. All patch clamp experiments were performed at 37 °C unless otherwise mentioned.

### 2.5. Immunofluorescence

Undifferentiated iPSCs cultures, trilineage differentiation cultures and cardiomyocyte cultures were fixed with 4% paraformaldehyde for 15 min and permeabilized by incubating in DPBS with 1% BSA and 0.1% Triton X-100 for 15 min at room temperature. iPSCs were labeled for pluripotent stem cell marker OCT3/4. Trilineage differentiation cultures were evaluated for markers of ectoderm (Pax6), mesoderm (Brachyury) and endoderm (AFP). Cardiomyocytes were evaluated for markers (cTnT and α-actinin). Samples were incubated in primary antibodies at 4 °C overnight, followed by incubation in secondary antibodies at room temperature for 60 min. Mouse and rabbit isotype controls (ThermoFisher Scientific, Waltham, MA, USA) were used in the control experiments. Nuclei were stained with Hoechst stain. Samples were studied using inverted fluorescent microscope (Olympus, Shinjuku, Tokyo, Japan).

### 2.6. Flow Cytometry

iPSC cultures and cardiomyocyte cultures were detached with ACCUTASE and trypsin, respectively. The fixation/permeabilization procedure was performed using BD Cytofix/Cytoperm kit (BD Biosciences, Franklin Lakes, NJ, USA) following manufacturer’s instructions. The purity of iPSC was determined by staining with Oct3/4, Nanog and SSEA-4 antibodies, while the purity of cardiomyocytes was determined with cTnT antibody (all from Miltenyi, Bergisch Gladbach, Germany). Samples were subjected to flow cytometry analysis (CytoFLEX, software: CytExpert; Beckman Coulter, Brea, CA, USA). Mouse and rabbit isotype controls (ThermoFisher Scientific, Waltham, MA, USA) were used in the control experiments.

### 2.7. STR Analysis

DNA was isolated using DNeasy Blood & Tissue Kit (QIAGEN, Düsseldorf, Germany) and amplified by PCR using STR Multi-Amplification Kit (PowerPlex 21D System; Promega, Madison, WI, USA) following manufacturer’s instructions. PCR products were assayed with 3100 DNA Analyzer (ThermoFisher Scientific, Waltham, MA, USA). Tested loci were: AMEL, D3S1358, D1S1656, D6S1043, D13S317, Penta E, D16S539, D18S51, D2S1338, CSF1PO, Penta D, TH01, vWA, D21S11, D7S820, D5S818, TPOX, D8S1179, D12S391, D19S433 and FGA. 

### 2.8. Karyotyping

iPSCs were treated with 10 μg/mL of Colcemid (ThermoFisher Scientific, Waltham, MA, USA) for 60 min at 37 °C. Subsequently, cells were dissociated with Accutase, treated with 0.075 M hypotonic KCl solution, and fixed with Carnoy’s fixative. Five metaphase spreads were prepared and examined by G-banding. 

### 2.9. Whole Exome Sequencing

DNA was isolated using DNeasy Blood & Tissue Kit (QIAGEN, Düsseldorf, Germany) and loci consisting of mutations were amplified using Q5 high-fidelity DNA polymerase (NEB). Sanger sequencing was conducted to validate the mutation. 

### 2.10. Statistical Analysis

Statistical analyses were performed using GraphPad Prism Software (GraphPad Software, CA, USA) and R Studio. Data are presented as mean ± S.D. Student’s *t*-test and Welch’s *t*-test were used for statistical analysis and *p*-values < 0.05 were considered significant. 

### 2.11. Information on Material Suppliers

Information on material suppliers and material catalog numbers is available in Table S4.

## 3. Results

### 3.1. Patient Diagnosis and Treatment

The patient first suffered palpitation without known cause in 2012, whereas only symptom-relieving treatments were given. The patient was hospitalized in 2016 due to palpitation, seizure, chest tightness and more severe symptoms including arrhythmia and ventricular fibrillation (Table S1). Emergency treatments including cardiopulmonary resuscitation were conducted. The patient was later diagnosed with long QT syndrome with arrhythmia. Conventional medications including beta-blockers (Metoprolol and Esmolol) were not effective in treating symptoms. Only after ICD implantation and additional medication (increased dose of Esmolol and adding Propranolol to the treatment regime), the patient stabilized and was later discharged. After discharging, Propranolol was subscribed for disease management.

In 2017, the patient was hospitalized multiple times due to relapses of LQTS symptoms similar to 2016. Frequent ICD discharging and arrhythmic storm were also recorded, indicating the condition of the patient could be worsening. In additional to beta-blockers (Metoprolol, Esmolol and Propranolol of increased dose), anti-arrhythmia agents (Amiodarone and Benazepril) were also employed so symptoms were barely managed. Based on the fact that the patient was suffering from continual yet severe LQTS symptoms and conventional medications were not effective, peripheral blood was acquired to establish a patient-specific iPSC line for disease modelling and drug screening. The ultimate aim is to identify alternative therapeutic agents that might enhance the clinical outcome. Please also refer to Table S1 for details of the patient’s medical history.

### 3.2. Derivation of Long QT Patient-Specific iPSC Line FAHXMUi001-A

**Figure 1:**
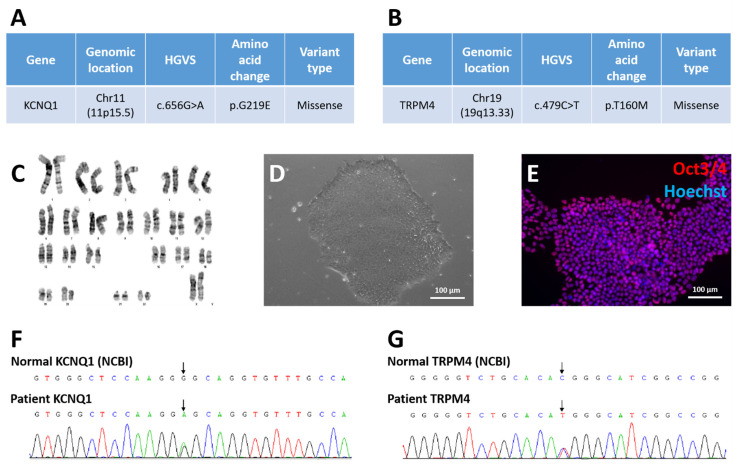
Derivation of LQTS-iPSC line FAHXMUi001-A from patient PBMCs. Heterozygous KCNQ1 (**A**) and TRPM4 (**B**) mutations were identified in patient PBMCs through WES. iPSCs derived from patient PBMCs demonstrated normal 46 XX karyotype (**C**), typical colony morphology of pluripotent stem cells (**D**) and were positive for pluripotent stem cell marker Oct3/4 (**E**). By using WES, heterozygous KCNQ1 (**F**) and TRPM4 (**G**) mutations were identified in FAHXMUi001-A cells.

Whole exome sequencing (WES) of patient peripheral blood mononuclear cells (PBMCs) revealed a heterozygous KCNQ1 c.656G>A mutation with p.G219E (Substitution—Missense, position 219, G→E) amino acid change (Figure 1A) and a heterozygous TRPM4 c.479C>T mutation with p.T160M (Substitution—Missense, position 160, T→M) amino acid change (Figure 1B). 

The LQTS patient-specific iPSC line FAHXMUi001-A was derived from patient PBMCs with the use of CytoTune^®^ 2.0 Sendai Reprogramming Kit (Thermo Scientific). Four transcription factors, namely OCT3/4, SOX2, KLF4 and c-MYC, were utilized to foster reprogramming. G-banding showed that FAHXMUi001-A had normal 46 XX karyotype (Figure 1C). Cultured FAHXMUi001-A cells demonstrated typical pluripotent stem cell morphology (Figure 1D) and were found to be positive for pluripotent stem cell marker Oct3/4 when observed under fluorescence microscope (Figure 1E). To validate the KCNQ1 and TRPM4 mutations also present in the derived LQT-iPSCs, genomic DNA of FAHXMUi001-A was amplified and the sequencing result demonstrated heterozygous KCNQ1 c.656G>A mutation (Figure 1F) and heterozygous TRPM4 c.479C>T mutation (Figure 1G) equivalent to starting PBMCs. STR result of FAHXMUi001-A matched with the PBMC STR profile of the corresponding patient, indicating that FAHXMUi001-A was patient-specific (Figure S1). Control iPSCs derived from the healthy donor also demonstrated typical pluripotent stem cell morphology and were found to be positive for Oct3/4 (Figure S2). Isotype control images are included in Figure S3.

### 3.3. Pluripotency Characterization of FAHXMUi001-A

**Figure 2:**
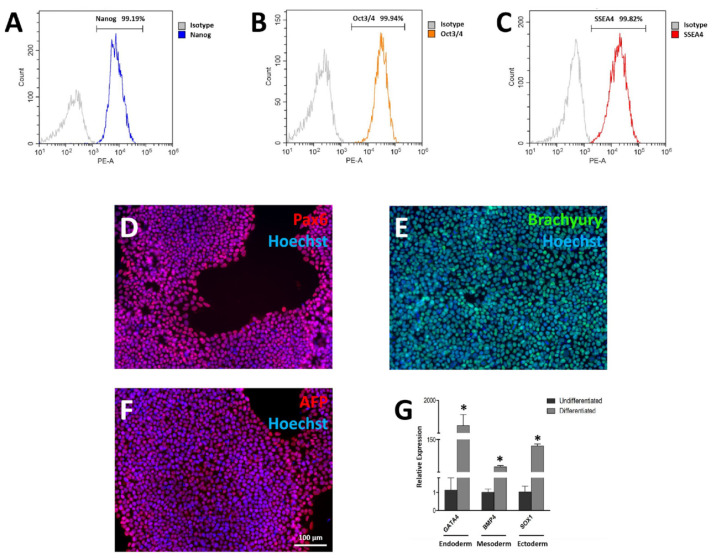
Characterization of LQTS-iPSC line FAHXMUi001-A. FAHXMUi001-A cells were demonstrated to be ≥ 95% positive for pluripotent stem cell markers Nanog (**A**), Oct3/4 (**B**) and SSEA (**C**) in flow cytometry analysis. Pax6 (**D**), Brachyury (**E**) and AFP (**F**) were detected by immunofluorescence, while Sox1, BMP4 and GATA4 were detected by RT-qPCR after FAHXMUi001-A cells were subjected to trilineage differentiation (**G**). Flow cytometry graphs and immunofluorescence images are representative samples. All experiments were of *n* = 3 or larger. * = *p* ≤ 0.005.

Expression of pluripotent stem cell markers and trilineage differentiation potential of FAHXMUi001-A were studied. Flow cytometry showed that FAHXMUi001-A cells at passage 10 were highly positive for pluripotent stem cell markers Nanog (Figure 2A), Oct3/4 (Figure 2B) and SSEA4 (Figure 2C). Through directed differentiation, FAHXMUi001-A cultures were successfully differentiated into cells representing the three germ layers, where expression of ectoderm markers Pax6 and Sox1, mesoderm markers Brachyury and BMP4 and endoderm markers AFP and GATA4 were determined by immunofluorescence imaging and qPCR analysis, respectively (Figure 2D–G). Control iPSCs at passage 10 were also found to be highly positive for pluripotent stem cell markers and capable of trilineage differentiation (Figure S2). These results indicated that FAHXMUi001-A cells and control iPSCs were pluripotent and capable of differentiating into all three germ layers in vitro. To confirm reproducibility of reprogramming, extra iPSC clones were derived from both the patient and the healthy donor. Please refer to Figure S4 (patient) and Figure S5 (healthy donor).

### 3.4. Cardiomyocyte Differentiation of FAHXMUi001-A

**Figure 3:**
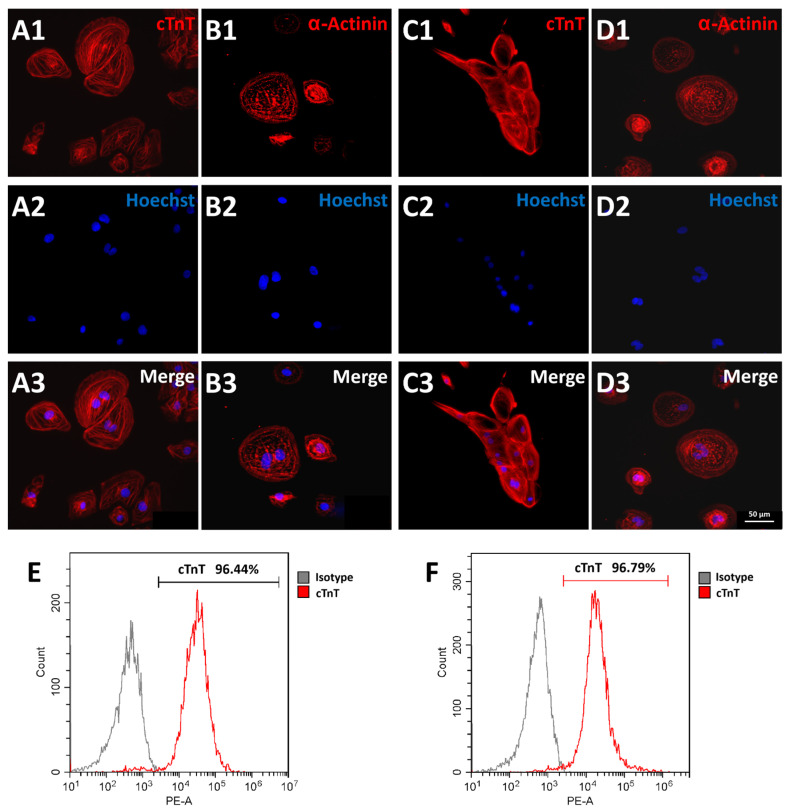
Characterization of Control and LQTS Cardiomyocytes. Ctrl-CMs and LQTS-CMs were positive for cardiomyocyte markers cTnT ((**A1**–**A3**), Ctrl-CMs; (**C1**–**C3**), LQTS-CMs) and α-actinin ((**B1**–**B3**), Ctrl-CMs; (**D1**–**D3**), LQTS-CMs). Flow cytometry analysis suggested that both Ctrl-CM and LQTS-CM consisted of ≥95% cTnT positive cells ((**E**), Ctrl-CMs; (**F**), LQTS-CMs). Flow cytometry graphs and immunofluorescence images are representative samples. All experiments were of *n* = 3 or larger.

With the use of a specific method, Ctrl-iPSCs and LQTS-iPSCs were successfully differentiated into cardiomyocytes. At approximately 8 to 10 days after differentiation treatment started, spontaneous contraction could be observed (Supplementary Video S1). Cryopreserved and subsequently thawed cardiomyocytes were subjected to immunofluorescence and flow cytometry analysis, respectively. For immunofluorescence, cardiomyocytes were cultured on cover slips and labeled against cardiomyocyte marker cardiac muscle troponin T (cTnT) and α-actinin. Ctrl-CMs and LQTS-CMs were positive for cardiomyocyte markers cTnT (Figure 3A,C) and α-actinin (Figure 3B,D). Segmental organization of cTnT and α-actinin was observed in both Ctrl-CMs and LQTS-CMs. For flow cytometry analysis, cardiomyocytes labeled against cTnT were analyzed. Results revealed cTnT percent positive ≥ 90% in both Ctrl-CMS (Figure 3E) and LQTS-CMs (Figure 3F). These results indicate that cardiomyocytes derived from iPSCs were of high purity and were suitable for subsequent experiments. Isotype control images were included in Figure S3. To confirm reproducibility of cardiomyocyte differentiation, extra iPSC clones were derived from both the patient and the healthy donor and were differentiated into cardiomyocytes. Please refer to Figure S6.

### 3.5. Drug Treatment and Electrophysiological Analysis

**Figure 4:**
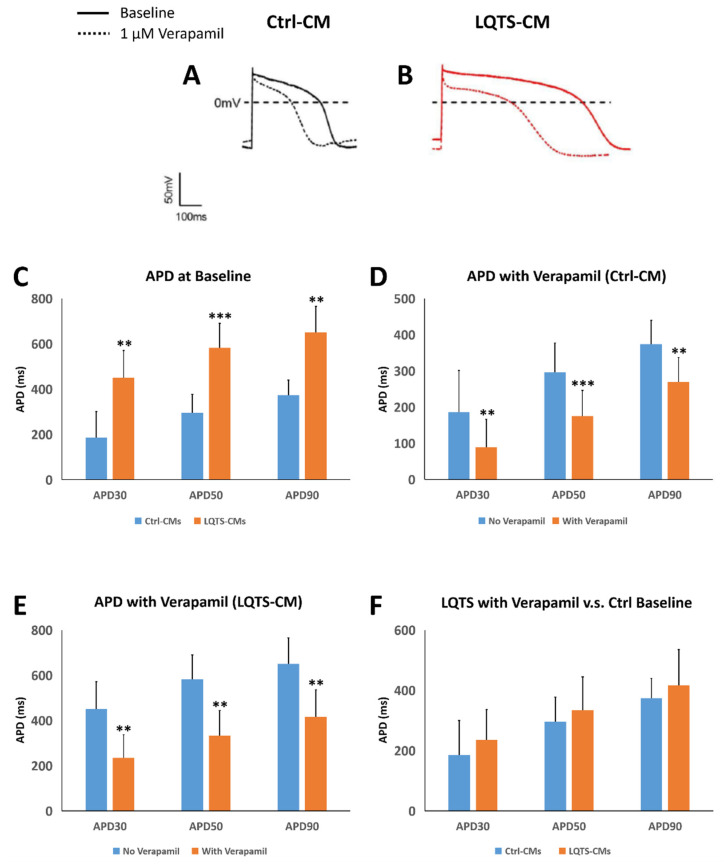
Electrophysiological Analysis of Verapamil Treatment. Whole cell patch clamp revealed APD profiles of Ctrl-CMs (**A**) and LQTS-CMs (**B**), respectively. Without any treatment, LQTS-CMs demonstrated significantly longer QT intervals than Ctrl-CMs in terms of APD_30_, APD_50_ and APD_90_ (**C**). Treatment of 1 μM verapamil resulted in significant reduction of APD_30_, APD_50_ and APD_90_ in Ctrl-CMs (**D**) and LQTS-CMs (**E**). APD_30_, APD_50_ and APD_90_ of LQTS-CMs treated with 1 μM verapamil demonstrated no statistically significant difference when compared to APD values of Ctrl-CMs at baseline (**F**). Cardiomyocyte action potential graphs and immunofluorescence images are representative samples. All experiments were of *n* = 3 or larger. ** = *p* ≤ 0.01; *** = *p* ≤ 0.005.

Before drug treatment, Ctrl-CMs and LQTS-CMs cultured on cover slips were analyzed using whole cell patch clamp to identify QT intervals by measuring action potential duration 30, 50 and 90 (regarded as APD_30_, APD_50_ and APD_90_). LQTS-CMs demonstrated significantly longer QT intervals than Ctrl-CMs in terms of APD_30_, APD_50_ and APD_90_ (Figure 4A–C). After being treated with 1 μM verapamil, significant reductions in QT intervals in terms of APD_30_, APD_50_ and APD_90_ were recorded in both Ctrl-CMs (Figure 4A,D) and LQTS-CMs (Figure 4B,E), respectively. When LQTS-CMs were treated with verapamil, their APD_30_, APD_50_ and APD_90_ were no longer statistically different from Ctrl-CMs at baseline (Figure 4F). Such a result suggests that verapamil treatment effectively reduced QT interval of LQTS-CMs to a level similar to that of normal cardiomyocytes. Please refer to Supplementary Table S2 for APD recording of individual cells.

**Figure 5:**
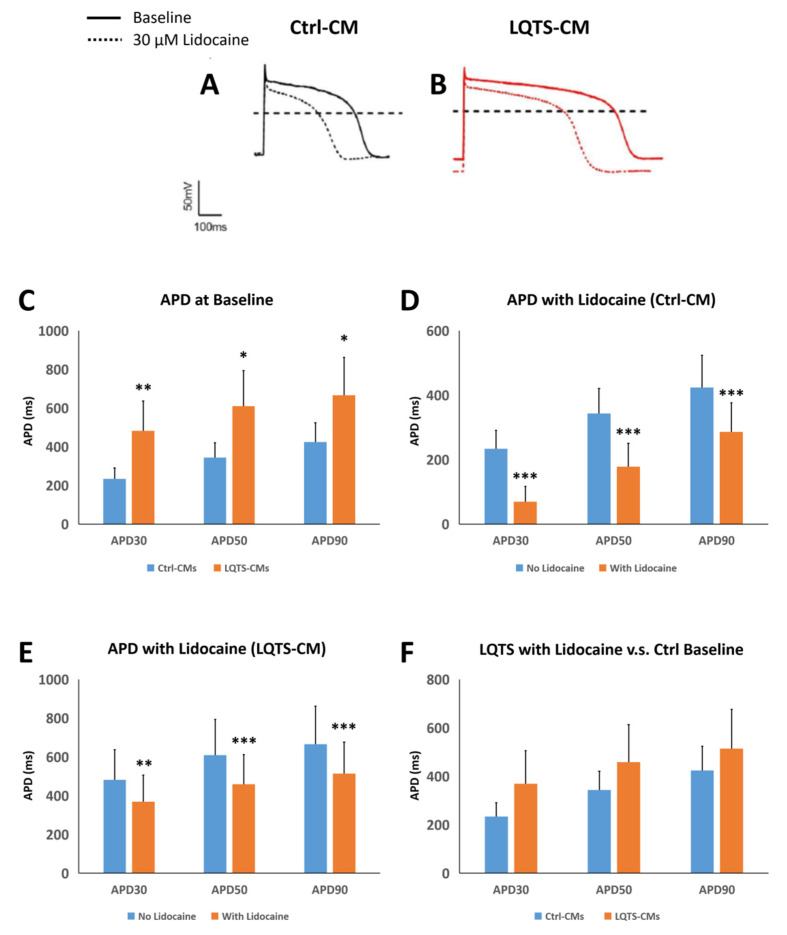
Electrophysiological Analysis of Lidocaine Treatment. Whole cell patch clamp revealed APD profiles of Ctrl-CMs (**A**) and LQTS-CMs (**B**), respectively. Without any treatment, LQTS-CMs demonstrated significantly longer QT intervals than Ctrl-CMs in terms of APD_30_, APD_50_ and APD_90_ (**C**). Treatment of 30 μM lidocaine resulted in significant reduction of APD_30_, APD_50_ and APD_90_ in Ctrl-CMs (**D**) and LQTS-CMs (**E**). APD_30_, APD_50_ and APD_90_ of LQTS-CMs treated with 30 μM lidocaine demonstrated no statistically significant difference when compared to APD values of Ctrl-CMs at baseline (**F**). Cardiomyocyte action potential graphs and immunofluorescence images are representative samples. All experiments were of *n* = 3 or larger. * = *p* ≤ 0.05; ** = *p* ≤ 0.01; *** = *p* ≤ 0.005.

Treatment of 30 μM lidocaine resulted in significant decreases in QT intervals in terms of APD_30_, APD_50_ and APD_90_ in both Ctrl-CMs (Figure 5A,D) and LQTS-CMs (Figure 5B,E). Similar to the verapamil scenario, LQTS-CMs treated with lidocaine demonstrated no statistically significant differences in terms of APD_30_, APD_50_ and APD_90_ when compared to Ctrl-CMs at baseline (Figure 5F). Such a result suggests that lidocaine treatment effectively reduced QT interval of LQTS-CMs to a level similar to that of normal cardiomyocytes. Please refer to Supplementary Table S3 for APD recording of individual cells.

**Figure 6:**
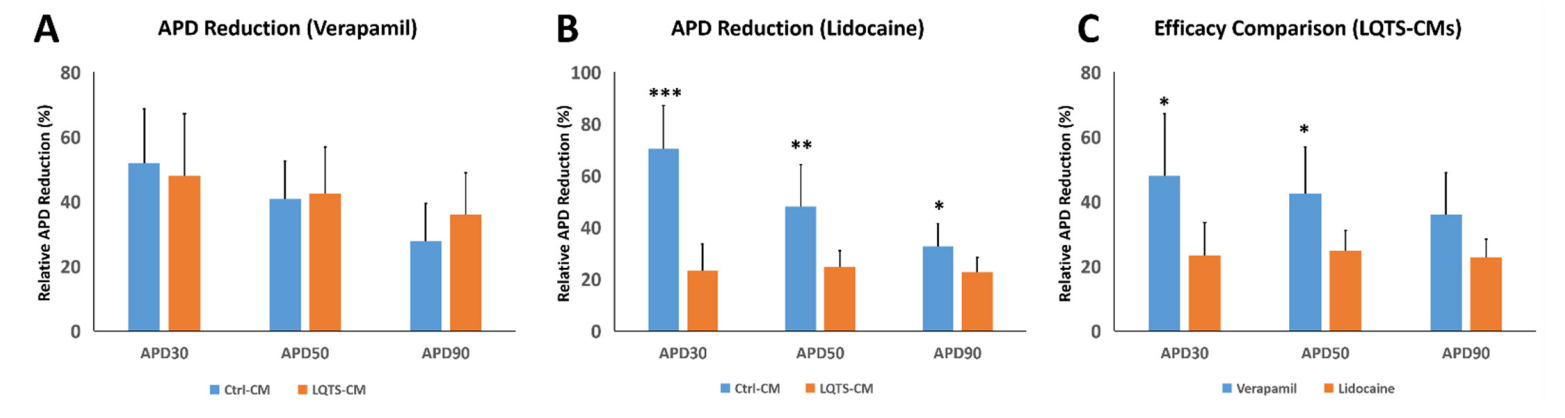
Comparison of QT Interval Shortening Efficacy between Verapamil and Lidocaine. After verapamil treatment, Ctrl-CMs and LQTS-CMs demonstrated no significant difference in relative QT interval shortening (**A**). After lidocaine treatment, however, QT interval reduction in Ctrl-CMs was significantly larger than LQTS-CMs (**B**). In LQTS-CMs, verapamil treatment achieved a significantly larger relative QT interval reduction in terms of APD_30_ and APD_90_ than lidocaine (**C**). All experiments were of *n* = 3 or larger. * = *p* ≤ 0.05; ** = *p* ≤ 0.01; *** = *p* ≤ 0.005.

Effects of verapamil and lidocaine on QT interval reduction were further analyzed in detail. After verapamil treatment, relative reduction in QT intervals of Ctrl-CMs and LQTS-CMs demonstrated no significant difference (Figure 6A). In contrast, after lidocaine treatment, Ctrl-CMs demonstrated larger relative reduction in QT interval than LQTS-CMs (Figure 6B). Relative reduction in QT interval in LQTS-CMs was also found to be larger after verapamil treatment than after lidocaine treatment (Figure 6C). These results suggest that for the KCNQ1/TRPM4 dual mutation scenario, verapamil is potentially a better alternative medication than lidocaine.

**Figure 7:**
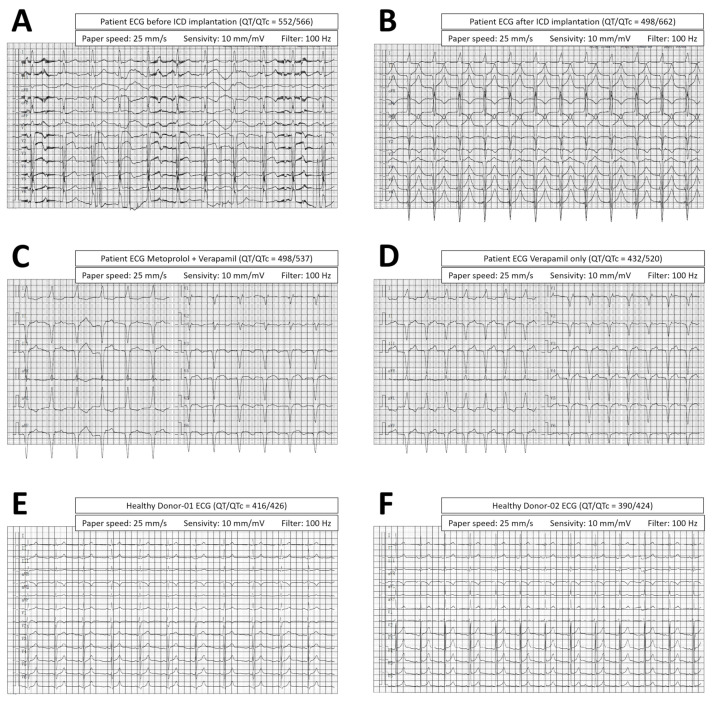
Effect of Verapamil on Patient ECG. Dual mutation patient demonstrated QT intervals of 552/566 and 498/662 before ICD implantation (**A**) and after ICD implantation (**B**), respectively. Patient QT intervals were found to be longer when compared to ECGs of healthy donor 01 ((**E**), QT/QTc = 416/426) and 02 ((**F**), QT/QTc = 390/424). Treatment using Metoprolol in combination with verapamil (**C**) or verapamil alone (**D**) both reduced QT interval and improved ECG wave form.

ECG of the dual mutation patient before ICD implantation (Figure 7A) and after ICD implantation but while receiving only conventional drugs (Figure 7B) demonstrated QT/QTc of 552/566 and 498/662, respectively. ECG before ICD implantation was poorly organized with twisted waves. After ICD implantation, wave form was improved but the QT interval remained long when compared to ECGs of healthy donor 01 (Figure 7E, QT/QTc = 416/426) and 02 (Figure 7F, QT/QTc = 390/424). Provided the KCNQ1/TRPM4 dual mutation patient was not responding well to conventional medications and suffered relapses of LQTS symptoms, verapamil (oral, 40 mg, 3 times per day) was added to the treatment regime. We first tried verapamil in combination with Metoprolol (oral, 23.75 mg, once per day). Treatment of Metoprolol in combination with verapamil resulted in further improved ECG wave form and a reduction in QT interval (Figure 7C, QT/QTc = 498/537). After removing Metoprolol from the treatment regime, we discovered that verapamil alone was sufficient to maintain a better ECG wave form and a reduced QT interval (Figure 7D, QT/QTc = 432/520). Upon using verapamil, the patient also demonstrated reduced frequency of ICD discharging (Table S1).

## 4. Discussion

With the use of iPSC technology, the current study successfully established a rare long QT syndrome disease model carrying a heterozygous KCNQ1 c.656G>A and a heterozygous TRPM4 c.479C>T mutation. To our knowledge, both these two mutations are newly discovered and the dual mutation scenario is also specific to the patient involved in the current study.

A major difficulty encountered in the treatment of this dual mutation LQTS patient is the ineffectiveness of conventional medications. Mainstream beta-blockers including Metoprolol, Esmolol and Propranolol were found effective, at least to a certain degree, in managing LQTS symptoms and preventing sudden cardiac death [6,7,8,31,32]. In the current study, however, the dual mutation patient did not respond well to Metoprolol and Esmolol. As a result, ICD implantation was performed and additional beta-blocker (Propranolol) and medication for the control of arrhythmia (i.e., Amiodarone and Benazepril [33,34]) were employed so disease manifestation could be barely managed. Still, ICD discharging was frequent and the patient suffered from seizures and severe symptoms such as arrhythmia storm. Such a subpar treatment outcome resulted in repeated hospitalization of the patient and poor quality of life.

By establishing a patient-specific dual mutation LQTS disease model based on iPSC technology, LQTS cardiomyocytes specific to the patient with significantly elongated QT interval were being derived in one year of time. Using manual patch clamp and mid- to low-throughput strategy, two potentially useful medications (i.e., verapamil and lidocaine) were successfully identified in the second year of the current study. Through the use of patient-specific iPSC disease model, the current study demonstrated that both verapamil and lidocaine are potential therapeutic agents useful in treating congenital LQTS by efficiently shortening the QT interval. Considering lidocaine is predominantly available in liquid dose for intravenous injection, oral dose verapamil was instead added to the treatment regime of the dual mutation patient for daily maintenance purposes. Adding verapamil to the treatment regime successfully reduced the frequency of ICD discharge, which in turn enhanced patient quality of life. ECG also indicated reduction in QT interval. Our result suggested that verapamil could be an effective drug for LQTS in certain conditions. More importantly, these clinical improvements highlighted the usefulness of patient-specific disease modelling in drug screening and tailoring personalized treatment strategy for not only congenital LQTS patients but also patients in rare disease scenarios where mainstream medications are found to be not effective. Once such patients have been identified, conventional treatments could be employed to at least delay disease progression. In the meantime, patient-specific iPSCs and corresponding disease-related functional cell type (cardiomyocytes in the case of LQTS) could be derived for drug screening. Based on drug screening results, the treatment strategy of the patient could be modified in a real-time manner to achieve a better clinical outcome. By streamlining processes of iPSC reprogramming and subsequent differentiation into cardiomyocytes or other functional cell types, time needed to establish a personalized drug screening platform could be shortened so better drugs could be identified as soon as possible. By substituting manual patch clamp with mid-throughput screening systems (e.g., automatic patch clamping) or high-throughput screening systems (e.g., multi-electrode array (MEA)), the efficiency of drug screening could also be further improved. In addition, effectiveness of drugs might be dependent on the genotype of congenital disease patients. Such characteristics further the need for disease models harbouring the mutations of the patient. We therefore consider the personalized, iPSC-based drug screening platform presented in the current study a good strategy to identify better alternative therapeutic agents for a particular patient with his/her unique disease conditions.

As mentioned, the patient involved in the current study is a carrier of a heterozygous KCNQ1 c.656G>A and a heterozygous TRPM4 c.479C>T mutation. Such a unique dual mutation combination might have contributed to the ineffectiveness of mainstream LQTS medications. Treatment efficacy of beta-blockers depends on the genotype of congenital LQTS and possibly this can also apply to other potential therapeutic agents [32]. Both heterozygous KCNQ1 c.656G>A and heterozygous TRPM4 c.479C>T are newly identified mutations. Heterozygous KCNQ1 c.656G>A mutation resulted in p.G219E (Substitution—Missense, position 219, G→E) amino acid change, which is located in the S3–S4 linker of the voltage sensor domain [35]. Mutation in the voltage sensor domain is known to induce KCNQ1 channel dysfunction, resulting in reduced outward potassium trafficking (I_KS_), diminished net outward current during the plateau phase and therefore longer APD and LQTS [36,37]. The heterozygous TRPM4 c.479C>T mutation causing p.T160M (Substitution—Missense, position 160, T→M) amino acid change is located in the N-terminus domain. Mutations in this domain could be correlated to slower calcium-dependent deactivation of the TRPM4 channel, which results in increased sodium influx, increased membrane potential and prolonged APD [38,39]. The exact mechanisms underlining LQTS correlated to the KCNQ1/TRPM4 dual mutation are yet to be defined. However, based on knowledge from literature, KCNQ1 mutation and TRPM4 mutation could be synergistic in increasing membrane potential, prolonging APD and finally contributing to long QT with severe clinical symptoms observed in the patient.

Verapamil is a calcium channel blocker known to reduce QT interval [23]. Blockade of calcium channel and subsequent reduction of calcium influx could have prevented the cardiomyocyte membrane potential from remaining positive and therefore promoted repolarization and shortened QT interval. Lidocaine, on the other hand, might have played a more complicated role. Lidocaine is a sodium channel blocker also capable of inhibiting TRPM7 [40]. However, lidocaine is also reported to inhibit potassium channels [41,42]. Lidocaine could have inhibited sodium channels, prevented excessive sodium influx, prevented delay in repolarization and therefore shortened QT interval. Simultaneously, lidocaine could have also inhibited potassium channels and resulted in delayed potassium outflux, which in turn delayed repolarization. In addition, the possibility of lidocaine inhibiting TRPM4 directly cannot be ruled out. Given that verapamil and lidocaine probably shortened QT interval through different mechanisms and verapamil demonstrated better efficacy in QT interval reduction, verapamil could be considered a better alternative medication for LQTS treatment than lidocaine.

## 5. Conclusions

Through the establishment of LQTS patient-specific iPSC line FAHXMUi001-A and subsequent cardiomyocyte differentiation, the current study offers an efficient pathway towards modelling of rare variants of LQTS and potentially other cardiovascular diseases. Drug screening based on cardiomyocytes derived from FAHXMUi001-A successfully identified verapamil and lidocaine as alternative therapeutic agents for the treatment of a rare KCNQ1/TRPM4 dual mutation long QT syndrome patient. The fact that efficacy of medication-based treatment of congenital LQTS depends on genotype further highlights the usefulness of the approach of the current study. Finally, results of the current study suggest that verapamil and lidocaine could be considered potential alternatives for LQTS treatment especially when first-line beta-blockers cannot manage disease manifestation. Further study, however, is needed to decipher not only mechanisms of action of verapamil and lidocaine but also pathogenic mechanisms underlining LQTS manifestation in the dual mutation patient.

## Data Availability

Datasets of the current study are available from the corresponding authors upon request.

## Supplementary Materials

The following are available online at https://www.mdpi.com/article/10.3390/cells11162495/s1, Figure S1: STR Analysis of Patient-Specific iPSC Line FAHXMUi001-A, Figure S2: Ctrl-iPSC Characterization, Figure S3: Immunofluorescence Isotype Controls, Figure S4: Characterization of LQTS-iPSCs Clone-02, Figure S5: Characterization of Ctrl-iPSCs from Healthy Donor-02, Figure S6: Characterization of Cardiomyocytes derived from LQTS-iPSCs Clone-02 and Ctrl-iPSCs from Healthy Donor-02, Table S1: Symptoms and Treatment Outcomes of Long QT Patient, Table S2: Verapamil Treatment of Cardiomyocytes—Individual Data Points, Table S3: Lidocaine Treatment of Cardiomyocytes—Individual Data Points, Table S4: Material Suppliers and Material Catalog Numbers.
