# Supplementary material for: In Vitro Drug Screening Using iPSC-Derived Cardiomyocytes of a Long QT-Syndrome Patient Carrying KCNQ1 & TRPM4 Dual Mutation: An Experimental Personalized Treatment
Source: Cells. 2022 Aug 11;11(16):2495. doi: 10.3390/cells11162495 (PMC9406448; doi:10.3390/cells11162495)
Supplement: Supplementary file 1 [file cells-11-02495-s001.zip › Supplementary Figure Legend (Fig. S1~S6).pdf]

**Figure S1: STR Analysis Results of Patient PBMC and LQTS-iPSCs**

Patient PBMC and LQTS-iPSCs were subjected to STR analysis with a total of 21 loci analyzed. Results suggested that results of PBMC (A) and LQTS-iPSCs (B) matched each other, indicating no contamination of non-donor cells.

**Figure S2: Characterization of Ctrl-iPSCs from Healthy Donor-01**

Ctrl-iPSCs demonstrated typical colony morphology of pluripotent stem cells (A) and were positive for pluripotent stem cell marker Oct3/4 (B). Ctrl-iPSCs also demonstrated ≥95% positive for pluripotent stem cell markers Nanog (C), Oct3/4 (D) and SSEA (E) in flow cytometry analysis. Pax6 (F), Brachyury (G) and AFP (H) were detected by immunofluorescence after Ctrl-iPSCs were subjected to trilineage differentiation.

**Figure S3: Isotype Controls of Immunofluorescence**

Mouse IgG and rabbit IgG isotype controls were employed in immunofluorescence analysis of iPSCs and cardiomyocytes to match corresponding antibodies used. Mouse and rabbit isotype controls yield no detectable fluorescence signal in iPSCs (A & B) and cardiomyocytes (C & D), suggesting that antibodies used in immunofluorescence analysis of iPSCs and cardiomyocytes were specific.

**Figure S4: Characterization of LQTS-iPSCs Clone-02**

LQTS-iPSCs clone-02 demonstrated typical colony morphology of pluripotent stem cells (A) and were positive for pluripotent stem cell marker Oct3/4 (B). LQTS-iPSCs clone-02 also demonstrated ≥95% positive for pluripotent stem cell markers Nanog (C), Oct3/4 (D) and SSEA (E) in flow cytometry analysis.

**Figure S5: Characterization of Ctrl-iPSCs from Healthy Donor-02**

Ctrl-iPSCs from healthy donor-02 demonstrated typical colony morphology of pluripotent stem cells (A) and were positive for pluripotent stem cell marker Oct3/4 (B). from healthy donor-02 also demonstrated ≥95% positive for pluripotent stem cell markers Nanog (C), Oct3/4 (D) and SSEA (E) in flow cytometry analysis.

**Figure S6: Characterization of Cardiomyocytes derived from LQTS-iPSCs Clone-02 and Ctrl-iPSCs from Healthy Donor-02**

LQTS-CMs from LQTS-iPSCs clone-02 and Ctrl-CMs from Ctrl-iPSCs of healthy donor-02 were positive for cardiomyocyte markers cTnT (A, LQTS-CMs; C, Ctrl-CMs) and $\alpha$-actinin (B, LQTS-CMs; D, Ctrl-CMs). Flow cytometry analysis suggested that both LQTS-CM and Ctrl-CM consisted of ≥95% cTnT positive cells (E, LQTS-CM; F, Ctrl-CM). Flow cytometry graphs and immunofluorescence images are representative samples.
