# Supplementary material for: In Vitro Drug Screening Using iPSC-Derived Cardiomyocytes of a Long QT-Syndrome Patient Carrying KCNQ1 & TRPM4 Dual Mutation: An Experimental Personalized Treatment
Source: Cells. 2022 Aug 11;11(16):2495. doi: 10.3390/cells11162495 (PMC9406448; doi:10.3390/cells11162495)
Supplement: Supplementary file 1 [file cells-11-02495-s001.zip › Supplementary Tables.pdf]

Supplementary Table S1: Symptoms and Treatment Outcomes of Dual Mutation Long QT Patient

Time	Symptoms	Treatments	Outcomes & Remarks
2012	Palpitation<br><br>Other symptoms like seizure or chest tightness were not observed	Symptom relief treatment only	LQTS diagnosis not yet made
Oct, 2016	Hospitalized with the following symptoms:<br>◆ Palpitation<br>◆ Seizure<br>◆ Chest tightness<br>◆ Sweating<br>◆ Arrhythmia<br>◆ Ventricular fibrillation	Emergency treatment<br>◆ Cardiopulmonary resuscitation<br>◆ Defibrillation<br><br>Medication<br>◆ Metoprolol (23.75 mg → 47.5 mg per day oral)<br>◆ Esmolol (1 g in 50 mL, 2 mL/hr IV)<br><br>Device Treatment<br>◆ ICD implantation<br><br>Medication after ICD Implantation<br>◆ Propranolol (20 mg, 3 times per day oral)<br><br>Medication after patient discharge<br>◆ Propranolol (20 mg, 3 times per day oral)	LQTS diagnosis was made<br><br>Initial treatment using Metoprolol and Esmolol was not effective even after increasing the dose of Metoprolol<br><br>Patient stabilized only after ICD implantation and adding Propranolol to the treatment regime
Feb, 2017	Hospitalized for multiple times with the following symptoms:<br>◆ Palpitation<br>◆ Seizure<br>◆ Chest tightness<br>◆ Sweating<br>◆ Arrhythmic storm<br>◆ Loss of consciousness<br><br>Frequent ICD discharging and arrhythmic storm recorded	Medication<br>◆ Metoprolol (47.5 mg per day oral)<br>◆ Esmolol (1 g in 45 mL, 5 mL/hr IV)<br>◆ Propranolol (40 mg, 3 times per day oral)<br>◆ Amiodarone (450 mg in 41 mL, 9 mL/hr, IV)<br><br>Medication after patient discharge<br>◆ Propranolol	Patient quality of life was severely deteriorated due to symptom relapse, frequent ICD discharging and arrhythmic storm

		(40 mg, 3 times per day oral)	
Aug, 2017	Hospitalized for multiple times with the following symptoms:<br>◆ Palpitation<br>◆ Seizure<br>◆ Chest tightness<br><br>ICD discharging recorded	Medication<br>◆ Metoprolol (47.5 mg per day oral)<br>◆ Esmolol (1 g in 45 mL, 5 mL/hr IV)<br>◆ Propranolol (40 mg, 3 times per day oral)<br>◆ Amiodarone (450 mg in 41 mL, 9 mL/hr, IV)<br><br>Device Treatment<br>◆ Optimization of ICD operating parameters<br><br>Medication after patient discharge<br>◆ Propranolol (40 mg, 3 times per day oral)<br>◆ Benazepril (10 mg daily)	Patient quality of life was severely deteriorated due to symptom relapse, ICD discharging
Oct, 2017	Hospitalized for multiple times with the following symptoms:<br>◆ Palpitation<br>◆ Seizure<br>◆ Chest tightness<br><br>Frequent ICD discharging recorded	Medication<br>◆ Metoprolol (47.5 mg per day oral)<br>◆ Esmolol (1 g in 45 mL, 5 mL/hr IV)<br>◆ Propranolol (60 mg, 3 times per day oral)<br><br>Medication after patient discharge<br>◆ Propranolol (60 mg, 3 times per day oral)	Patient quality of life was severely deteriorated due to symptom relapse, ICD discharging
2018 onward	LQTS symptoms relapse similar to 2017	Verapamil (40 mg, 3 times per day, oral) added to treatment regime by 2018	Symptom better controlled and ICD discharging frequency reduced since the use of Verapamil<br><br>QT interval reduced as demonstrated by ECG

Supplementary Table S2: Verapamil Treatment of Cardiomyocytes - Individual Data Points

Group		Cell	APD30	APD50	APD90
Healthy donor	Control	01	256.40	322.00	367.36
		02	340.56	418.43	472.29
		03	164.20	263.80	339.52
		04	119.98	275.18	392.99
		05	48.97	199.60	295.52
		**Mean**	**186.02**	**295.80**	**373.54**
		**SD**	**114.52**	**81.28**	**65.98**
	Verapamil	01	104.59	183.28	243.41
		02	210.00	272.96	311.28
		03	78.51	154.93	239.80
		04	47.17	187.33	362.60
		05	7.59	76.34	190.96
		**Mean**	**89.57**	**174.97**	**269.61**
		**SD**	**76.44**	**70.63**	**67.35**
Patient	Control	01	425.57	569.99	646.40
		02	316.75	504.23	571.40
		03	377.15	464.65	511.28
		04	513.79	634.79	723.94
		05	621.77	735.95	797.57
		**Mean**	**451.01**	**581.92**	**650.12**
		**SD**	**119.61**	**107.76**	**114.76**
	Verapamil	01	353.74	425.36	506.52
		02	98.74	175.98	244.38
		03	188.54	276.18	341.75
		04	221.18	352.40	462.75
		05	313.05	440.58	524.45
		**Mean**	**235.05**	**334.10**	**415.97**
		**SD**	**101.37**	**109.95**	**119.49**

All APD values are in ms.

Supplementary Table S3: Lidocaine Treatment of Cardiomyocytes - Individual Data Points

Group		Cell	APD30	APD50	APD90
Healthy donor	Control	01	290.34	419.52	530.32
		02	143.15	231.07	312.05
		03	249.75	295.72	333.26
		04	265.13	379.97	434.31
		05	219.30	388.39	509.05
		**Mean**	**233.53**	**342.93**	**423.80**
		**SD**	**56.78**	**77.53**	**99.26**
	Lidocaine	01	101.83	233.74	365.99
		02	8.36	53.30	158.07
		03	109.55	192.15	236.65
		04	98.04	223.16	297.64
		05	27.04	188.27	369.98
		**Mean**	**68.96**	**178.12**	**285.67**
		**SD**	**47.44**	**72.46**	**89.98**
Patient	Control	01	412.21	498.90	563.69
		02	326.59	475.33	520.26
		03	646.57	846.58	918.36
		04	649.58	769.36	832.34
		05	373.31	454.86	491.80
		**Mean**	**481.65**	**609.01**	**665.29**
		**SD**	**154.92**	**184.33**	**195.83**
	Lidocaine	01	250.17	325.44	387.68
		02	278.58	374.77	420.77
		03	474.57	619.35	686.91
		04	554.32	631.89	694.20
		05	288.35	341.56	381.44
		**Mean**	**369.20**	**458.60**	**514.20**
		**SD**	**136.28**	**153.56**	**161.70**

All APD values are in ms.

Supplementary Table S4: Material Suppliers and Material Catalog Numbers

No.	Material Name & Purpose	Supplier	Outcomes & Remarks
01	Vacutainer CPT	BD Biosciences, Franklin Lakes, NJ, USA	362753
02	CytoTune-iPS 2.0 Sendai Reprogramming Kit	ThermoFisher Scientific, Waltham, MA, USA,	A16517
03	mTeSR1 medium	Stemcell Technologies	85857
04	Matrigel	Corning, Corning, New York, USA	354230
05	Accutase	ThermoFisher Scientific, Waltham, MA, USA	1110501
06	STEMdiff™ Trilineage Differentiation Kit	Stemcell Technologies	05230
07	RPMI1640	ThermoFisher Scientific, Waltham, MA, USA	11875093
08	B27 no insulin	ThermoFisher Scientific, Waltham, MA, USA	A1895601
09	B27	ThermoFisher Scientific, Waltham, MA, USA	17504044
10	CHIR99021	Sigma-Aldrich, Burlington, MA, USA	SML1046
11	IWR-1	Sigma-Aldrich, Burlington, MA, USA	I0161
12	FBS	ThermoFisher Scientific, Waltham, MA, USA	26140079
13	Trypsin EDTA	ThermoFisher Scientific, Waltham, MA, USA	25200056
14	CryoStor CS10	BioLife Solution	210102
15	Verapamil	Sigma-Aldrich, Burlington, MA, USA	V4629
16	Lidocaine	Sigma-Aldrich, Burlington, MA, USA	L7757
17	Anti-Oct3/4 antibody	Abcam, Cambridge, UK	ab181557
18	Donkey anti-Mouse IgG (H+L) Highly Cross-Adsorbed Secondary Antibody,Alexa Fluor 546	ThermoFisher Scientific, Waltham, MA, USA	A10036
19	PE Mouse anti-SSEA4（Clone MC813-70(RUO)）	BD Biosciences, Franklin Lakes, NJ, USA	560128
20	PE Mouse IgG3, κ Isotype Control（Clone A112-3(RUO)）	BD Biosciences, Franklin Lakes, NJ, USA	559926
21	PE Mouse anti-Oct3/4（Clone 40/Oct-3(RUO)）	BD Biosciences, Franklin Lakes, NJ, USA	560186
22	PE Mouse IgG1, κ Isotype Control（Clone MOPC-21(RUO)）	BD Biosciences, Franklin Lakes, NJ, USA	559320
21	PE Mouse anti-human Nanog（Clone N31-355(RUO)）	BD Biosciences, Franklin Lakes, NJ, USA	560873
22	PE Mouse IgG1, κ Isotype Control（Clone MOPC-21(RUO)）	BD Biosciences, Franklin Lakes, NJ, USA	554680

23	Anti-alpha 1 Fetoprotein (AFP-01)	Abcam, Cambridge, UK	ab3980
24	Brachyury Rabbit mAb	Cell Signaling Technologies, Danvers, MA, USA	81694
25	Alexa Fluor® 488 Mouse anti-Human Pax-6（Clone O18-1330(RUO)）	BD Biosciences, Franklin Lakes, NJ, USA	561664
26	Donkey anti-Mouse IgG (H+L) Highly Cross-Adsorbed Secondary Antibody,Alexa Fluor 546	ThermoFisher Scientific, Waltham, MA, USA	A10036
27	Donkey anti-Rabbit IgG (H+L) Highly Cross-Adsorbed Secondary Antibody,Alexa Fluor 488	ThermoFisher Scientific, Waltham, MA, USA	A21206
28	Anti-cTnT	Abcam, Cambridge, UK	ab45932
29	Anti-alpha Actinin	Abcam, Cambridge, UK	ab68194
30	Donkey anti-Mouse IgG (H+L) Highly Cross-Adsorbed Secondary Antibody,Alexa Fluor 546	ThermoFisher Scientific, Waltham, MA, USA	A10036
31	Hoechst 33342	ThermoFisher Scientific, Waltham, MA, USA	H3570
32	PE Mouse Anti-Cardiac Troponin T	ThermoFisher Scientific, Waltham, MA, USA	564767
33	PE Mouse IgG1, κ Isotype Control	BD Biosciences, Franklin Lakes, NJ, USA	554680
