# Supplementary figures and images for: In Vitro Drug Screening Using iPSC-Derived Cardiomyocytes of a Long QT-Syndrome Patient Carrying KCNQ1 & TRPM4 Dual Mutation: An Experimental Personalized Treatment
Source: Cells. 2022 Aug 11;11(16):2495. doi: 10.3390/cells11162495 (PMC9406448; doi:10.3390/cells11162495)

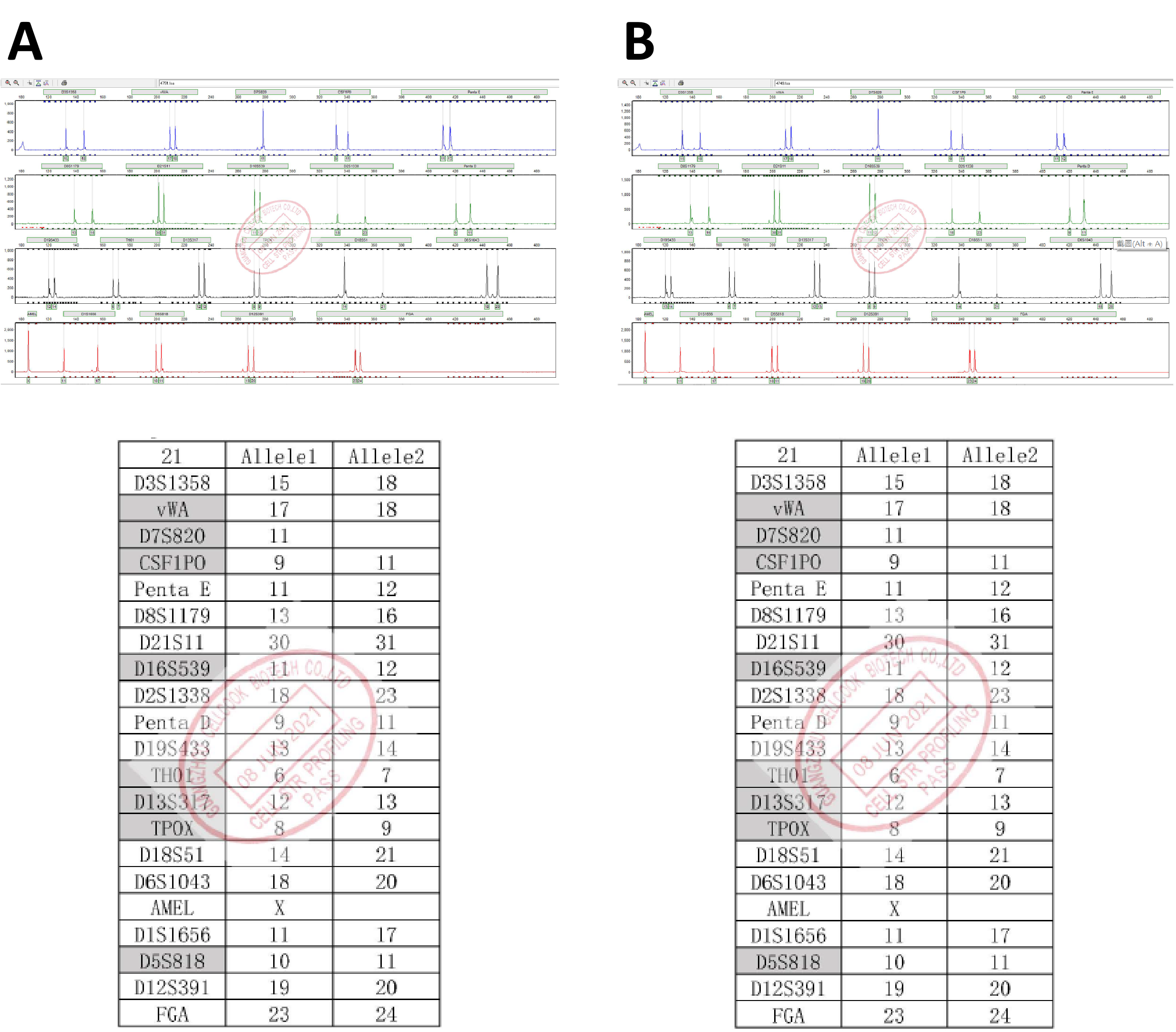

Supplement: Supplementary file 1 [file cells-11-02495-s001.zip › Figure S1.tif]

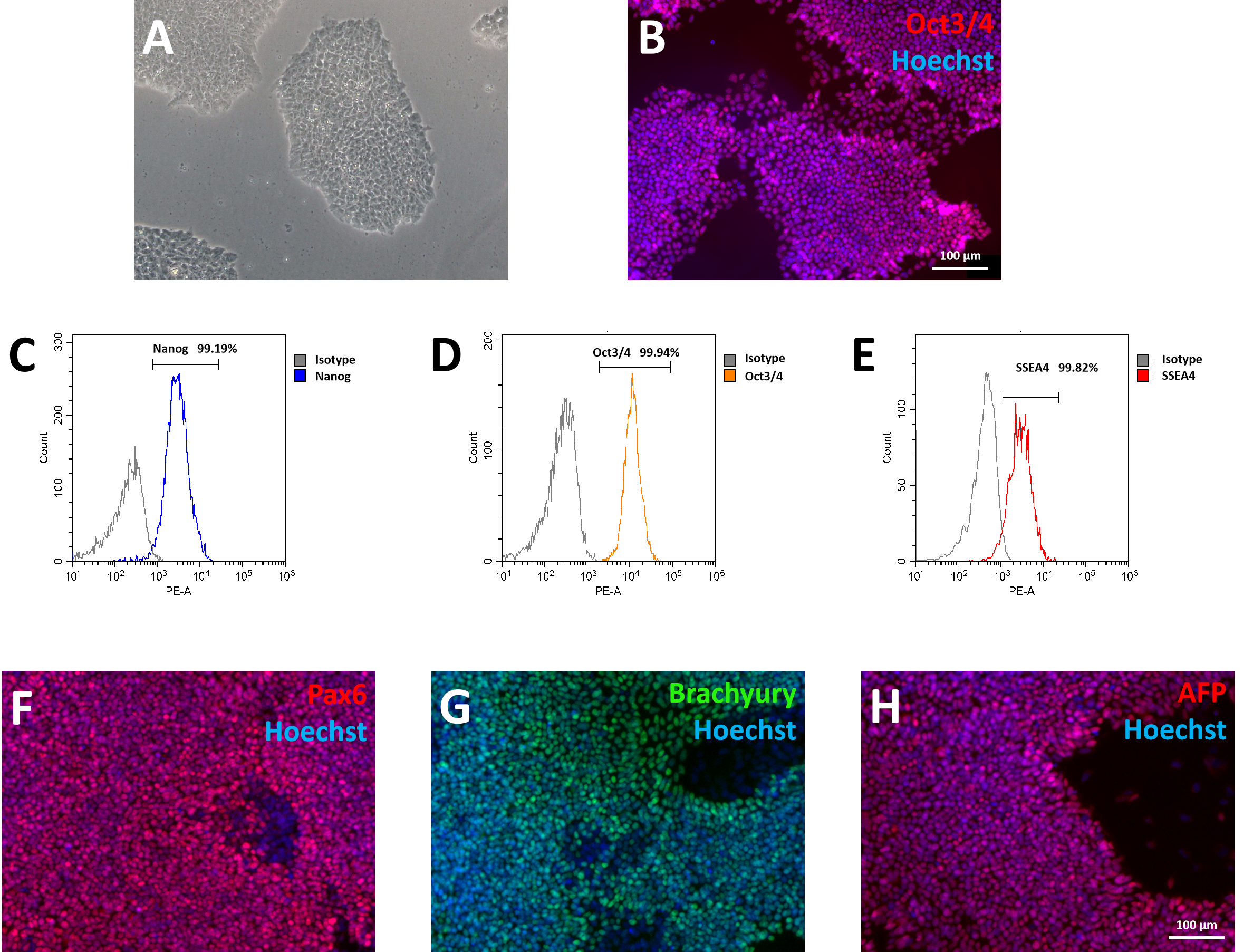

Supplement: Supplementary file 1 [file cells-11-02495-s001.zip › Figure S2.tif]

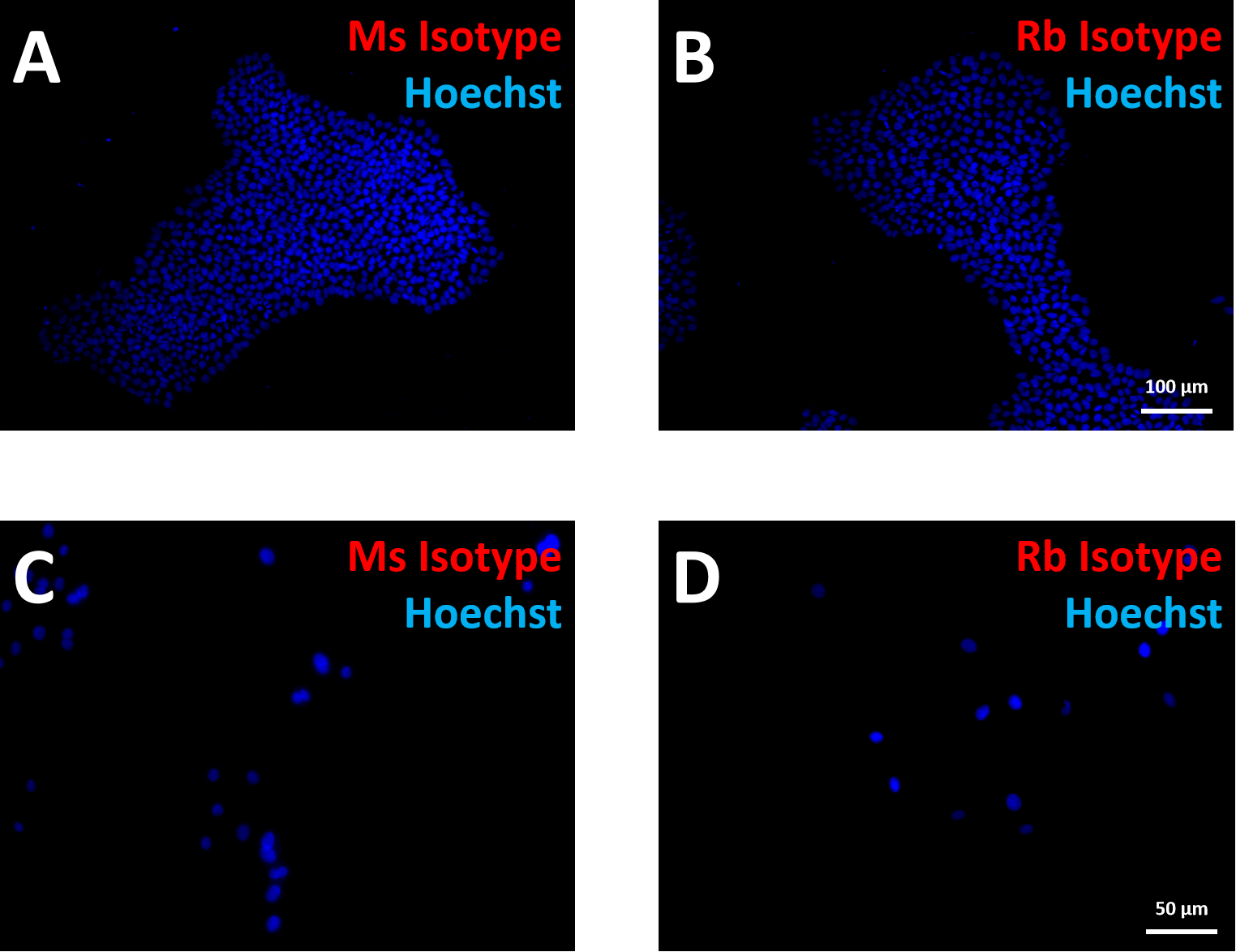

Supplement: Supplementary file 1 [file cells-11-02495-s001.zip › Figure S3.tif]

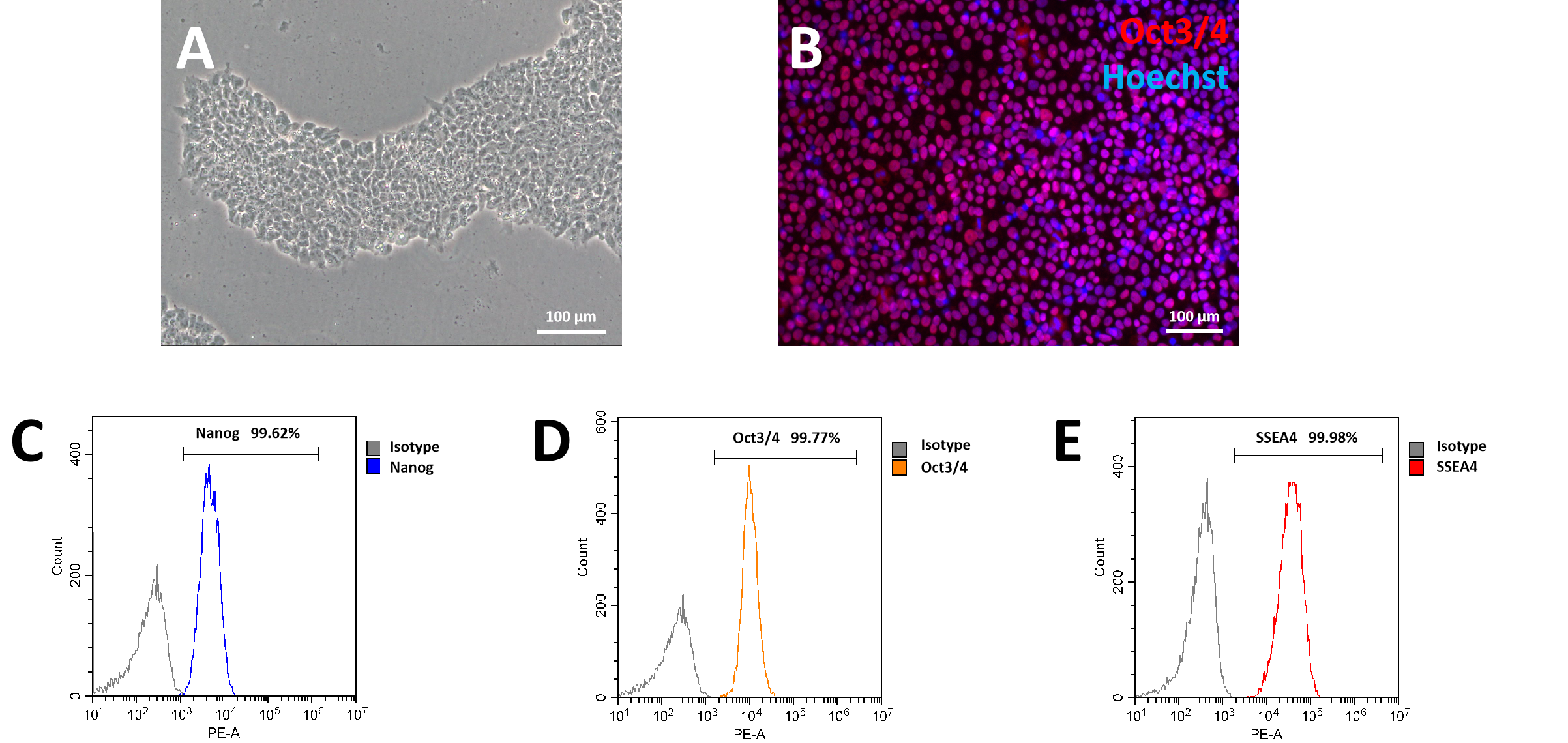

Supplement: Supplementary file 1 [file cells-11-02495-s001.zip › Figure S4.tif]

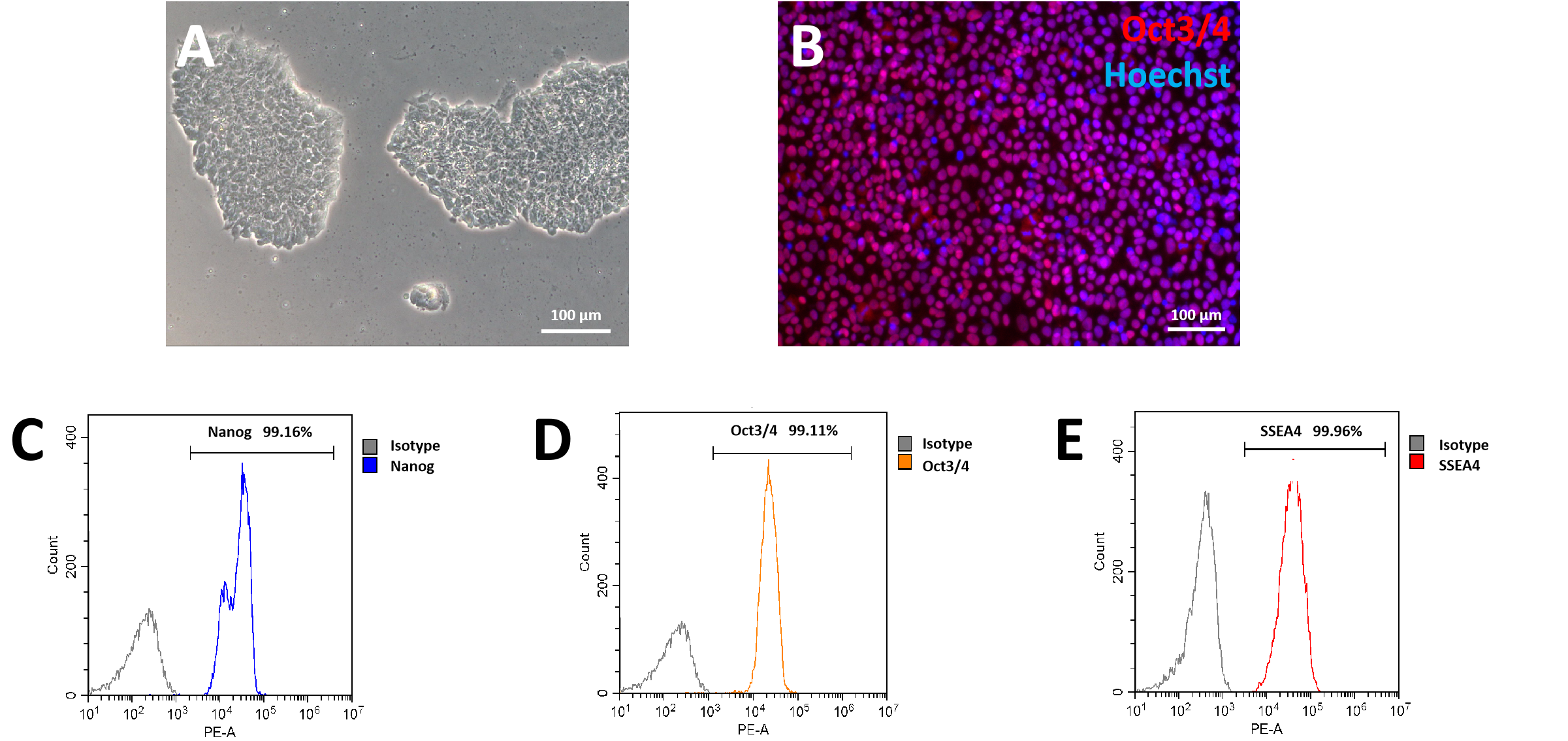

Supplement: Supplementary file 1 [file cells-11-02495-s001.zip › Figure S5.tif]

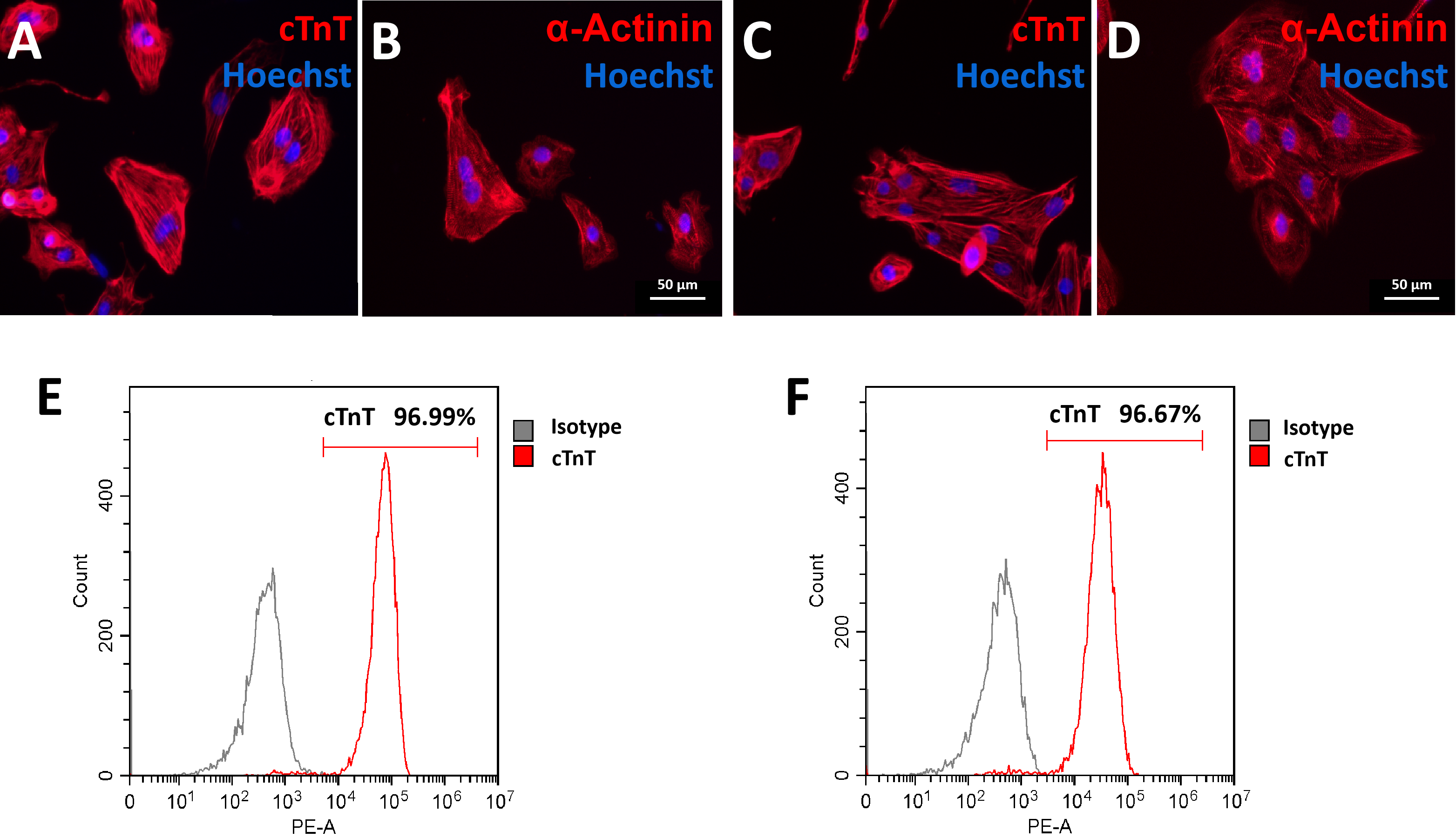

Supplement: Supplementary file 1 [file cells-11-02495-s001.zip › Figure S6.tif]
